# The Impacts of Lifetime Violence on Women's Current Sexual Health

**DOI:** 10.1089/whr.2023.0089

**Published:** 2024-02-01

**Authors:** Ayşe Güler, Megan K. Maas, Kristen P. Mark, Nurlan Kussainov, Katie Schill, Ann L. Coker

**Affiliations:** ^1^ Center for Research on Violence Against Women, Department of Biostatistics, College of Public Health, University of Kentucky, Lexington, Kentucky, USA.; ^2^ Department of Human Development & Family Studies, College of Social Science, Michigan State University, East Lansing, Michigan, USA.; ^3^ Department of Family Medicine and Community Health, Eli Coleman Institute for Sexual and Gender Health, University of Minnesota, University of Minnesota Medical School, Minneapolis, Minnesota, USA.; ^4^ Center for Clinical and Translational Science, University of Kentucky, Lexington, Kentucky, USA.; ^5^ Department of Obstetrics and Gynecology, University of Kentucky College of Medicine, Lexington, Kentucky, USA.

**Keywords:** lifetime violence, intimate partner violence, child abuse, sexual health

## Abstract

**Background:**

Intimate partner violence (IPV), nonpartner sexual violence (SV), child sexual and physical abuse, and neglect have detrimental impacts on women's reproductive and sexual health. More empirical studies are needed to investigate the negative impacts of lifetime violence, including physical or sexual child abuse, nonpartner SV, physical, sexual, and psychological IPV on women's sexual health to better understand long-term impacts from IPV and physical or sexual child abuse.

**Materials and Methods:**

We used data from Wellness, Health and You, an ongoing health registry. A total of 1,213 women were included in data analysis. Our aim was to investigate the associations between lifetime IPV, nonpartner SV, child abuse, and women's current sexual health defined using Patient-Reported Outcomes Measurement Information System (PROMIS) measures of sexual health (*e.g*., sexual satisfaction, interest, and functioning), sexual assertiveness, female sexual subjectivity, and use of online resources to address sexual needs. Multivariate analysis of covariance was used to investigate demographic factors (*e.g*., age and current relationship) as potential correlates of current sexual health.

**Results:**

Women with lifetime experiences of physical, sexual, or psychological IPV, nonpartner SV, and child physical or sexual abuse reported lower sexual satisfaction compared to women with no history of lifetime violence (*p* < 0.0001). However, lifetime violence was not correlated with sexual interest, sexual functioning, sexual subjectivity, nor sexual assertiveness.

**Conclusion:**

Lifetime experiences of violence (*i.e*., IPV, nonpartner SV, child abuse) are associated with poorer sexual health. Asking questions about past sexual and physical violence/abuse in ways that support disclosure is important toward improving women's physical and sexual health and wellbeing.

## Introduction

Globally, nearly 30% of women have experienced either physical and/or sexual intimate partner violence (IPV) or nonpartner sexual violence (SV) in their lifetime.^1^ In the United States, over 36.4% of women experience sexual and physical IPV, stalking, and psychological aggression by an intimate partner in their lifetime.^2^ These experiences often occur earlier, with more than one in seven children exposed to physical, sexual, and emotional abuse or neglect (Center for Disease Control and Prevention, 2022), and nearly 20% of women experience sexual abuse before 18 years of age.^3,4^

IPV, nonpartner SV, and child physical abuse and neglect, specifically child sexual abuse (CSA), have detrimental impacts on women's reproductive and sexual health. Physical and sexual IPV are associated with sexually transmitted infections, including HIV/AIDS infection, unintended pregnancy, pelvic inflammatory disease, cervical cancer, urinary tract infections, sexual dysfunction, and dyspareunia (*i.e*., pain with intercourse).^1,5–9^

Specifically, female sexual dysfunction involves distress over problems with sexual desire, arousal, orgasm, or sexual pain.^4,10^ Results of a systematic review show that physical and sexual IPV are associated with experiencing lower sexual desire, less pleasurable sex, sexual dissatisfaction, sexual pain, and chronic pelvic pain among women.^11^ Recent systematic review results indicate that sexual and emotional IPV are associated with dyspareunia, vaginal dryness, and vaginal irritation.^12^ Women with a history of SV and severe clinical symptoms of posttraumatic stress disorder (PTSD) experience vaginal dryness, vaginal irritation, and dyspareunia.^13^

Similarly, CSA is associated with sexual dysfunction, including sexual avoidance, lower sexual satisfaction, vaginismus, dyspareunia, and sexual anxiety in adult women.^14,15^ Women with a history of CSA may experience negative sexual self-concept, and higher prevalence of sexual dysfunctions, including arousal disorder, orgasm issues, dyspareunia, and sexual dissatisfaction, compared to women with no experiences of CSA.^4,16–18^

There has been strong support for a biopsychosocial approach to sexual health that simultaneously considers physical, psychological, sociocultural, and interpersonal factors in sexual function.^19–21^ This complex interplay involves hormones such as testosterone that may be able to mediate sexual functioning.^22^ This becomes particularly important in the experience of trauma. For example, a condition of the hypothalamic pituitary ovary gland is often seen in patients after PTSD due to the inhibitory effect of increased cortisol level on hypothalamic pituitary ovary glad suppression.^23^ Therefore, a reduction of testosterone production, as observed in hypothalamic amenorrhea conditions, could have a negative impact on sexuality, particularly sexual desire and arousal.

Several mechanisms could explain different pathways between lifetime sexual or physical violence experienced as a child or an adult and negative sexual health outcomes. For instance, the biological mechanism and structural and functional changes in the brain may cause alterations in the hypothalamic-pituitary-adrenal (HPA) axis, as the primary system involved in the physiological stress response. It is well-established that the HPA axis is dysregulated by chronic stress.^24^ Experiencing higher levels of psychological distress caused by violence resulting from trauma may lead to dysregulations in the HPA axis.^25^ When the HPA-axis is dysregulated, individuals face difficulty with decision-making and often experience dissociative states that could interfere with sexual decision-making and enjoyment.

Therefore, posttraumatic symptomatology or traumatic stress symptoms (*e.g*., PTSD) from lifetime violence experience may act as a mediator between lifetime violence exposure and sexual dysfunction among women,^26,27^ and thereby women with a history of lifetime sexual or physical violence exposure are more likely to report poorer sexual health compared to women with no history of lifetime violence.

While prior studies demonstrate the association between sexual health problems and IPV and CSA, empirical studies are needed to investigate the negative impacts of lifetime violence, including physical or sexual child abuse, nonpartner SV, physical, sexual, and psychological IPV on women's sexual health to better understand long-term impacts from lifetime violence.

Our specific aim was to investigate the associations between lifetime IPV, nonpartner SV, child abuse, and women's current sexual health defined using Patient-Reported Outcomes Measurement Information System (PROMIS)^28^ measures of sexual health (*e.g*., sexual satisfaction, interest, and functioning), sexual assertiveness,^29^ female sexual subjectivity,^30^ and use of online resources to address sexual needs.

## Materials and Methods

We used data from Wellness, Health and You (WHY), an ongoing health registry which includes annual surveys from a convenience sample (*N* = 5,447) in a southern state in the United States. For the purposes of this analysis, longitudinal data were aggregated across survey years to identify any potential exposure to child abuse or adult physical or sexual or psychological IPV. Among those who completed the sexual health survey, only one record per participant was included in the current analysis. The design for this investigation was cross-sectional where the exposure to violence (*i.e*., child abuse or adult physical or sexual or psychological IPV or nonpartner SV) was collected retrospectively and sexual health was assessed related to the status of their sexual health at the time of data collection. The WHY research protocol has been approved by University of Kentucky Institutional Review Board (protocol no. 43533).

### Measurement

#### Lifetime interpersonal violence: adult IPV, adult nonpartner SV, child physical and/or sexual abuse

The following six lifetime violence experience questions (sexual, physical, or psychological IPV, adult nonpartner SV, and CSA or physical abuse) were based on items included in the Behavioral Risk Factor Surveillance System.^31^ Lifetime IPV exposure was measured using three questions that solicited a yes or no response: (1) sexual IPV “Has an intimate partner used force [like hitting, holding down, or using a weapon] to make you have sex [any sex act, not just intercourse]?”; (2) physical IPV “Has an intimate partner hit, kicked, punched, or otherwise hurt you?”; and (3) psychological IPV “Has an intimate partner made serious threats, tried to control you, or humiliated you on purpose.”

Adult nonpartner SV was measured with the following question that solicited a yes or no response: “Has anyone, other than an intimate partner or anyone else, used force—such as hitting, holding down, or using a weapon to make you have sex, any sex act, not just intercourse?.” Child physical and sexual abuse were measured using the following BRFSS questions that solicited a yes or no response: (1) “Did a parent, stepparent, or guardian ever hit, kick, punch, or otherwise hurt you?; and (2) Did a parent, stepparent, or guardian ever make you have sex [any sex act, not just intercourse] by using force or by threatening to harm you or someone close to you?.” Because SV has been associated with poorer sexual health, we used a hierarchical approach to prioritized SV, measured as adult partner SV, adult nonpartner SV, or CSA.

Specifically, (1) those experiencing any form of SV, regardless of physical or psychosocial violence, were grouped together, (2) those experiencing physical violence as an adult or child, yet no SV, were grouped regardless of psychologic IPV, (3) and those experiencing psychologic IPV “alone” were grouped together. For these three groups experiencing violence, the referent group were those not experiencing violence or abuse.

#### Sexual health measures (primary outcomes)

Three existing scales were used to measure indicators of sexual health. The scales included the PROMIS Sexual Health module, the Female Sexual Subjectivity Inventory (FSSI), and the Sexual Assertiveness Questionnaire (SAQ-12). The PROMIS sexual health module^31,32^ was adapted to include three subscales: sexual satisfaction (5 items, 1 factor, variance explained [VE] = 3.63, Cronbach's *α* = 0.903), sexual interest (5 items, 1 factor VE = 3.19, *α* = 0.87), and sexual functioning (5 items, 1 factor, VE = 3.43, *α* = 0.78). Our adaptation was changing the time reference period from the past 7 days to the past month depicted in Supplementary A, Supplementary Table S1 for PROMIS sexual health measures.

A 4-item subscale of the FSSI^30^ was included as an indicator of a woman's sense of entitlement to sexual pleasure and safety from a partner (1 Factor, *n* = 1,191, VE = 3.29; *α* = 0.928) see Supplementary A, Supplementary Table S2. Twelve items from the SAQ^29^ were included as measures of communications about sexual initiation and satisfaction (*α* = 0.866; 12 items, Range: 0–55) 1 Factor, *n* = 1,191, VE = 5.58). See Supplementary A, Supplementary Table S3.

A 4-item measure assessing the frequency participants visited or used online resources to address sexual needs was created for this research (M.K.M., A.L.C.). The items addressed visits to websites dedicated to (1) sexual health information such as contraception, sexually transmitted infections, and pregnancy; (2) sexual pleasure information such as orgasm technique, mindfulness during sex, and mutual pleasure, but would not be considered pornography; (3) pornography such as depictions of genitals touching, mouths on genitals, and genitals being penetrated; (4) dating apps or websites such as Tinder, Grinder, Bumble, and Match. Response options ranged from Daily ( = 10), Weekly ( = 4), Once in the Past Month ( = 1), and Never or not applicable ( = 0) (1 factor, VE = 1.79, *α* = 0.575).

Demographic data collected included current age (based on the participant's most recently completed survey), race (self-reported, then grouped as White, Black, Asian, Hispanic, or Other), sex assigned at birth (restricted to female for the current analyses), current employment status, highest education, (based on the participant's most recently completed survey). See Table 2 for the demographic attribute categorizations.

### Statistical analysis

#### Sample size

A total of 1,274 WHY participants completed the sexual health module. Eleven men were excluded from analysis for not being women and 21 women were excluded due to missing responses for adult violence (*n* = 21) and 29 with incomplete responses for sexual health items outside the scale range of 4–25. A total of 1,213 women with no missing data on adult violence or sexual health measures were included in the final analysis.

#### Confounder assessment

Demographic attributes of WHY participants were investigated as correlates of lifetime violence experiences (primary exposure). Test of differences in proportions for demographics by lifetime violence rates were used (Chi-square, Table 2). Because current sexual health was the primary outcome, as assessed using the three PROMIS subscales, the FSSI, and SAQ-12, we opted to evaluate current demographic attributes as potential confounders of association between lifetime violence and sexual health. Multivariate analysis of covariance (MANCOVA) was used to investigate demographic factors (*e.g*., age and current relationship) as potential correlates of current sexual health. Demographic factors associated with violence were included in subsequent models as potential confounders. To investigate the association between timing (child or adult), types of violence exposure (*e.g*., physical or sexual or psychological IPV or nonpartner SV, three sets of measures were included in MANCOVA.

#### Hypothesis testing

The *p*-values associated with Wilks' Lambda for MANCOVA were significant (*p* < 0.05) indicating that these five dependent variables were correlated and use of MANCOVA was deemed appropriate. The following indicators of current sexual health were included in one MANCOVA model as dependent variables: (1) PROMIS sexual interest, (2) PROMIS sexual function, (3) FSSI, (4) SAQ-12, and (5) use of online resources to address sexual needs. Because the PROMIS measure of satisfaction was asked for those currently in a relationship, this outcome was included in a separate model of participants who were in a relationship at the time of data collection.

The following covariates were included in MANCOVA or ANCOVA models: age groups (*e.g*., ages 18–34, 35–44, 45–54, 55–64, 65+), marital status (currently married, divorced, or separated, widowed, never married), education (< college, college graduate, or post baccalaureate), current relationship status (in a relationship or not), and current employment status (employed or not employed). All analyses were conducted using SAS 9.4.

The interpersonal violence exposure measures were included in separate MANCOVA analyses to determine the associated between the form and timing of interpersonal violence and the six indicators of sexual health.

## Results

Among the 1,213 women included in this analysis, 41.9% (*n* = 508) disclosed lifetime abuse as a child (16.9%) or in adulthood (IPV: 31.3%, nonpartner SV: 11.8%; Table 1). Psychological IPV (21.4%) was the most commonly disclosed form of violence or abuse, and Psychological IPV alone without sexual or physical IPV was 10%. Among women experiencing lifetime IPV, 9% disclosed sexual IPV and 18.6% physical IPV. While 11.8% disclosed nonpartner SV (*i.e*., SV by someone other than a partner), 10.9% of women disclosed physical abuse, and 8.2% sexual abuse as a child; and 20% experienced both two forms of sexual abuse as a child or in adulthood (partner or nonpartner).

**Table 1.** Frequency of Lifetime Partner Violence, Sexual Violence, and Childhood Sexual and/or Physical Abuse (*N* = 1,213)

Form of violence^a^	***n***	Percent
Any physical, sexual, or psychological violence	508	41.9
Any sexual abuse or forced sex (child or adult)	243	20.0
Any physical violence/abuse (child or adult)	299	24.6
Any psychological abuse (measured only in adulthood)	260	21.4
In childhood (of 1,044 responses)		
Any physical abuse or physically forced sex (sexual abuse)	176^a^	16.9^a^
Any physical abuse	114^a^	10.9^a^
Any sexual abuse (child_sex_abuse = 86)	86^a^	8.2^a^
Missing	219	
As an adult (IPV)		
Partner physical, sexual, or psychological violence/abuse	380	31.3
Sexual IPV	109	9.0
Physical IPV	226	18.6
Psychological IPV	260	21.4
Psychological abuse alone (no sexual or physical IPV)	121	10.0
Nonpartner SV (Adult_SV)	143	11.8
CSA or adult SV (partner or nonpartner)	243	20.0
Adult or child physical violence/abuse	74	6.1
Psychological IPV alone	143	11.8
No violence in adult or childhood	750	61.8

^a^ Form of violence.

CSA, child sexual abuse; IPV, intimate partner violence; SV, sexual violence.

In an attempt to identify potential confounders for the association between lifetime violence or abuse and current sexual health indicators, lifetime violence rates were correlated with available demographic characteristics. Depicted in Table 2, lifetime violence rates were higher among WHY participants who had less education or were divorced or separated, and were older. Based on this analysis of potential confounders, only age was correlated with lifetime violence (*p* < 0.008). Current sexual relationship status, and education were additionally included in subsequent modeling as strong correlates of sexual satisfaction.

**Table 2.** Demographics by Lifetime Violence Experienced (*N* = 1,213)

	Within demographic characteristic (***n*** = 1,213)		With lifetime sexual, physical, or psychological abuse within demographic attribute	
	***n***	Percent	***n***	Percent
Age groups			χ^2^ = 15.53, df = 5, *p* = 0.0008	
18–34	108	8.9	31	28.6
35–44	171	14.1	64	37.7
45–54	200	16.5	97	48.5
55–64	251	20.7	108	42.9
65–74	330	27.2	151	45.8
75+	153	12.6	57	37.1
Race			χ^2^ = 1.29, df = 1, *p* = NS	
White or Caucasian	1,167	96.2	485	41.9
Black or African American	46	3.8	23	50.0
Education				
Highest educational attainment			χ^2^ = 5.40, df = 2, *p* = 0.07	
<college graduate	321	26.5	152	47.4
College graduate	350	28.9	141	40.3
Graduate or professional degree	542	44.7	215	39.7
Current marital status			χ^2^ = 3.76, df = 3, *p* = 0.05	
Married or living as married	806	66.4	346	41.6
Separated or divorced	180	14.8	107	59.4
Widowed	68	5.6	28	41.2
Single, never married	159	13.1	80	50.6
			χ^2^ = 0.77, df = 1, *p* = NS	
Currently in a relationship	829	68.3	340	41.1
Not currently in a relationship	384	31.7	168	43.8

NS, nonsignificant.

As hypothesized, lifetime sexual and physical violence experiences, defined hierarchically to prioritize lifetime SV, then physical violence, as a child or an adult, and finally, psychological IPV, were associated with lower current sexual satisfaction (*p* < 0.0001, see Table 3, 1st column). In general, lifetime SV was associated with lower sexual satisfaction for those experiencing SV as a child or adult (IPV) (*p* ≤ 0.0001). This pattern held across all interpersonal violence experiences in adulthood, yet, not childhood. Lifetime SV, IPV, or child abuse was not correlated with sexual interest, sexual function, sexual subjectivity, nor sexual assertiveness. Experiencing lifetime physical or sexual IPV or psychological IPV or nonpartner SV or child abuse was not associated with an increased use of online resources for sexual health.

**Table 3.** Multivariate Analysis of Covariance Results Assessing Lifetime Interpersonal Violence and Current Sexual Health Indicators (Sexual Satisfaction, Interest, and Function), Use of Online Resources, Sexual Subjectivity, and Sexual Assertiveness (*N* = 1,213)

	PROMIS sexual health measures						Sexual subjectivity^d^ Adjusted^*^ Mean (SE), ***t***-test		Sexual assertiveness^e^ Adjusted^*^ Mean (SE), ***t***-test		Use of online resources for sexual needs^f^ Adjusted^*^ Mean (SE), ***t***- test	
**Violence or abuse in adult or childhood**	Satisfaction^*^^,a^ Adjusted^**^ **Mean (SE), *t*-test**		**Interest^b^** Adjusted^**^ **Mean (SE), *t*-test**		**Function^c^** Adjusted^**^ **Mean (SE), *t*-test**							
Lifetime SV, IPV, or CA (*n* = 508, *n* = 373^*^)	15.84 (0.35)	−3.98, *p* < 0.0001	15.40 (0.20)	−0.40, *p =* NS	17.06 (0.22)	−1.77, *p =* 0.08	5.98 (0.13)	−1.43, *p =* NS	25.37 (0.53)	1.67, *p =* 0.09	0.86 (0.09)	1.24, *p =* NS
No violence (*n* = 704, 529^*^)	17.68 (0.30)	Ref.	15.51 (0.17)	Ref.	17.58 (0.19)	Ref.	6.23 (0.11)	Ref.	24.22 (0.45)	Ref.	0.71 (0.08)	Ref.
Lifetime violence (hierarchical^***^)												
Lifetime SV (*n* = 243, 172^*^)	15.77 (0.52)	−3.18, *p =* 0.002	15.64 (0.29)	0.38, *p =* NS	17.10 (0.32)	−1.28, *p =* NS	5.92 (0.19)	−1.36, *p =* NS	25.73 (0.75)	1.73, *p =* 0.08	0.96 (0.13)	1.66, *p =* 0.10
Lifetime Physical violence (*n* = 164,120^*^)	15.19 (0.63)	−3.58, *p =* 0.0004	15.18 (0.36)	−0.83, *p =* NS	17.45 (0.40)	−0.30, *p =* NS	5.71 (0.24)	−2.01, *p =* 0.04	25.01 (0.92)	0.77, *p =* NS	0.67 (0.16)	−0.22, *p =* NS
Psychological IPV alone (*n* = 101, 81^*^)	16.92 (0.76)	−0.94, *p =* NS	15.20 (0.45)	−0.64, *p =* NS	16.36 (0.50)	−2.30, *p =* 0.02	6.54 (0.30)	0.96, *p =* NS	25.05 (1.16)	0.67, *p =* NS	0.90 (0.20)	0.89, *p =* NS
No violence (*n* = 704, 529^*^)	17.68 (0.30)	Ref.	15.51 (0.17)	Ref.	17.58 (0.19)	Ref.	6.23 (0.11)	Ref.	24.22 (0.45)	Ref.	0.71 (0.08)	Ref.
Child abuse^***^												
CSA (*n* = 86, *n* = 56^*^)	15.19 (0.93)	−1.99, *p =* 0.05	15.53 (0.48)	0.16, *p =* NS	17.12 (0.53)	−0.39, *p =* NS	6.36 (0.32)	0.63, *p =* NS	27.84 (1.25)	2.40, *p =* 0.02	0.98 (0.20)	1.60, *p =* NS
Child physical abuse (*n* = 90, *n* = 66^*^)	15.69 (0.86)	−1.58, *p =* NS	15.32 (0.47)	−0.27, *p =* NS	16.84 (0.52)	−0.90, *p =* NS	5.46 (0.32)	−2.05, *p =* 0.04	24.08 (1.23)	−0.45, *p =* NS	0.68 (0.20)	0.23, *p =* NS
No child abuse (*n* = 867, *n* = 659^*^)	17.12 (0.27)	Ref.	15.45 (0.15)	Ref.	17.33 (0.17)	Ref.	6.14 (0.10)	Ref.	24.66 (0.40)	Ref.	0.64 (0.06)	Ref.
Adult IPV^***^												
Sexual IPV (*n* = 109, *n* = 76^*^)	14.45 (0.78)	−3.72, *p =* 0.0002	15.61 (0.43)	0.40, *p =* NS	17.01 (0.48)	−0.93, *p =* NS	5.44 (0.29)	−2.49, *p =* 0.01	24.62 (1.12)	0.14, *p =* NS	0.91 (0.19)	0.66, *p =* NS
Physical IPV, no sexual IPV (*n* = 152, *n* = 104^*^)	15.05 (0.67)	−3.42, *p =* 0.0007	15.73 (0.37)	0.78, *p =* NS	17.49 (0.41)	0.02, *p =* NS	6.05 (0.24)	−0.56, *p =* NS	25.85 (0.96)	1.33, *p =* NS	0.68 (0.17)	−0.49, *p =* NS
Psychological IPV alone (*n* = 123, *n* = 98^*^)	16.84 (0.69)	−0.94, *p =* NS	15.33 (0.41)	−0.19, *p =* NS	16.74 (0.45)	−1.53, *p =* NS	6.35 (0.27)	0.51, *p =* NS	25.05 (1.06)	0.53, *p =* NS	0.75 (0.18)	−0.10, *p =* NS
No IPV (827, 623)	17.54 (0.27)	Ref.	15.42 (0.16)	Ref.	17.48 (0.18)	Ref.	6.20 (0.10)	Ref.	24.45 (0.41)	Ref.	0.77 (0.71)	Ref.
Adult non-partner SV												
Adult non-partner SV (*n* = 143, 101^*^)	15.72 (0.69)	−1.85, *p =* 0.06	15.28 (0.38)	−0.52, *p =* NS	17.20 (0.42)	−0.41, *p =* NS	6.13 (0.25)	0.02, *p =* NS	25.51 (0.98)	0.88, *p =* NS	0.90 (0.17)	0.78, *p =* NS
No adult SV (*n* = 1,069, 801^*^)	17.07 (0.24)	Ref.	15.49 (0.14)	Ref.	17.38 (0.15)	Ref.	6.12 (0.09)	Ref.	25.59 (0.36)	Ref.	0.76 (0.06)	Ref.
Number of violence forms (*n* = 1,202, 935^*^)												
≥3 (*n* = 112, *n* = 76^*^)	14.25 (0.78)	−4.06, *p* < 0.0001	15.20 (0.67)	0.59, *p =* NS	17.57 (0.28)	−1.03, *p =* NS	5.86 (0.28)	−1.22, *p =* NS	26.09 (1.74)	1.58, *p =* NS	0.0.98 (0.19)	1.04, *p =* NS
2 (*n* = 124, *n* = 89^*^)	14.93 (0.72)	−3.50, *p =* 0.0005	15.55 (0.40)	0.08, *p =* NS	17.82 (0.44)	0.38, *p =* NS	5.74 (0.27)	−1.68, *p =* NS	24.03 (1.05)	−0.18, *p =* NS	0.70 (0.19)	−0.32, *p =* NS
1 (*n* = 254, *n* = 207^*^)	16.85 (0.47)	−1.43, *p =* NS	15.22 (0.28)	−0.95, *p =* NS	16.78 (0.31)	−2.37, *p =* 0.02	6.13 (0.19)	−0.45, *p =* NS	25.74 (0.73)	1.78, *p =* 0.07	0.92 (0.13)	1.07, *p =* NS
0 (*n* = 722, *n* = 564^*^)	17.63 (0.29)	Ref.	15.53 (0.18)	Ref.	17.63 (0.19)	Ref.	6.23 (0.11)	Ref.	24.23 (0.43)	Ref.	0.76 (0.08)	Ref.

^*^ Modules adjusting for age, current relationship status (in a relationship or not), Education (<college, college graduate, postbaccalaureate), Wilks' Lambda = 0.989, *F* = 2.54, Num DF (outcome = 5 outcomes, 2 levels of violence) = 5, Density DF = 1,176, *p =* 0.03.

^**^ Analysis among those in a relationship, PROMIS sexual satisfaction alone (*n* = 897) adjusting for age, and education.

Wilks' Lambda = 0.983 *F* = 15.83 Num DF = 1 (1 outcome × 2 levels of violence), Density DF = 895, *p* < 0.0001.

^***^ Hierarchical categories define to prioritize SV, then physical violence, psychologic violence relative to no violence/abuse.

^a^ PROMIS sexual health, sexual satisfaction subscale: *α* = 0.902; 5 items; Range: 0–25; ↑score = ↑satisfaction.

^b^ PROMIS sexual health, sexual interest subscale, *α* = 0.874; 5 items, Range: 5–25; ↑score = ↑interest.

^c^ PROMIS sexual health, sexual function subscale, *α* = 0.816; 5 items, Range: 5–25; ↑score = ↑function.

^d^ Female sexual subjectivity inventory, subscale − expectation of partner to meet sexual needs, *α* = 0.923; 4 items, Range: 0–20 ↑score = ↑expectations.

^e^ Sexual assertiveness questionnaire, *α* = 0.895; 12 items, Range: 0–55, ↑score = ↑sexual assertiveness.

^f^ Use of online resources to address sexual needs, *α* = 0.576; 4 items, time per month, Range: 0–20; ↑score = ↑use.

CA, child abuse; DF, degrees of freedom; PROMIS, Patient-Reported Outcomes Measurement Information System; SE, standard error.

## Discussion

We found that lifetime sexual and physical violence was consistently associated with lower sexual satisfaction across all combinations of lifetime violence experienced as an adult or child. Since the initial call from scholars^11^ for research to address the impacts of IPV on women's current sexual health, there has been limited examination of the associations between negative sexual health outcomes and lifetime interpersonal violence (*i.e*., child physical and sexual abuse, adult nonpartner SV, and physical and sexual IPV and psychological IPV) among women. To our knowledge, this investigation into the current sexual health of women who have experienced lifetime sexual or physical violence or psychological IPV is unique in its size (*N* = 902 adult women) and in the comprehensive measures of lifetime violence and sexual health to include sexual interest, sexual satisfaction and functioning, sexual assertiveness, and female sexual subjectivity.

Lifetime experiences of sexual, physical, and psychological IPV are associated with poorer sexual health. Results of a recent study showed that women with lifetime experiences of emotional, physical, or sexual IPV reported increased levels of worry and stress related to their current sex life, and sexual dysfunctions, including problems with sexual desire, orgasm, and sexual pain.^33^ Consistent with prior work, women with lifetime interpersonal violence experience higher prevalence of sexual avoidance, less interest in sex, dyspareunia, sexual anxiety, sexual dissatisfaction and distress, and problems with arousal orgasm compared to women without a history of violence.^10,11,14,15,17,34–36^ Our findings show that women with lifetime experiences of physical, sexual or psychological IPV, nonpartner SV, and child physical or sexual abuse reported lower sexual satisfaction (41.9%) compared to women with no history of lifetime violence.

Although we found significant associations between lifetime experiences of physical or SV as an adult or child and current sexual satisfaction, and the extent to which women used online resources for their sexual health needs were not significantly associated with lifetime sexual or physical violence. Those in the process of recovering from physical or SV may not yet be interested in pursuing online resources for satisfying and pleasurable sexual experience. Future research is needed to expand our results and investigate the confounding factors related to lifetime experiences of physical or sexual IPV or psychological IPV and child physical and sexual abuse on positive sexuality and sexual assertiveness among women. While primary prevention of sexual and physical violence victimization is essential, investigating approaches to mitigating the effect of violence/abuse on current sexual health defined to include positive sexuality, sexual functioning, and satisfaction also remain important to improving sexual health and wellbeing.

Some qualitative research has explored sexual pleasure and wellbeing after experiencing sexual trauma. Specifically, one finding of this body of work was that empathetic partner responses to disclosure of prior sexual trauma that are respectful of boundaries and allowed the survivor to lead the conversation were helpful for finding space to explore satisfying and pleasurable sexual relationships postsexual trauma.^37^ In addition, there are several strategies that women have reported being effective in establishing a healthy and satisfying sex life after experiencing sexual trauma, such as engagement in self-care, building social support, or communicating with their partner as concerns arise.^38^

Moreover, the disclosure to a trusted partner in itself is helpful for healing improve symptoms or perceptions.^39^ Testing a multidimensional quantitative model that identifies the strongest predictors of sexual satisfaction and pleasure while healing from sexual trauma would be an ideal next step in this body of research. We observed greater use of online resources to address sexual needs among those who experienced violence in adulthood, yet, no association with seeking online resources for those experiencing violence in childhood. This observation provides support for discerning potentially different needs for healing based on whether the SV took place during childhood or adulthood as important distinction that can guide treatment of sexual trauma.

### Implications

Sexual health problems associated with lifetime experiences of physical and SV as an adult or child are extensive.^5^ Therefore, health care providers may ask questions about past or current experiences of sexual and physical violence/abuse in ways that support disclosure. This remains a unique and strategically important tool toward improving women's physical and sexual health and wellbeing. Health care providers may also ask patients not only about their sexual health but also their sexual interest and satisfaction as both are key to physical health and emotional connections with their partners.

### Limitations

Those participating in WHY are disproportionately middle-aged, well-educated women and may, therefore, not be representative of the general population or those who may be at greatest risk of nonpartner SV or IPV. Nonetheless, even in this more privileged population, rates of sexual and physical violence were comparable to national estimates,^2^ and the effect of lifetime sexual and physical violence were consistently associated with poorer current sexual satisfaction. All data were collected through self-reports using reliable measures as indicated by strong psychometric properties for the majority of the current sexual health indicators. Self-reported data can be biased; however, for both past violence and current sexual health, the research participant is the only valid source of these data.

The potential for differential misclassification of current sexual health given violence experiences is unlikely, as questions regarding past violence or abuse were asked in prior surveys often years before the sexual health module was launched in 2020. We may have underestimated the effect of past violence or abuse on current sexual health because we did not have data to characterize receipt of services to address CSA or physical abuse, adult physical or sexual or psychological IPV, or nonpartner SV. We were able to adjust our analyses for potential confounding factors, including education and age, which may be associated with receipt of services. We were limited to data from cisgender women in this analysis and, therefore, future research should investigate the impact of lifetime physical or SV or current sexual health among nonbinary, transgender, or genderqueer people in addition to cisgender men.

## Conclusions

Findings of this investigation highlight the importance of asking questions about lifetime experiences of interpersonal violence (*i.e*., child physical and sexual abuse, adult nonpartner SV, and physical and sexual and psychological IPV) to improve positive sexual health outcomes. Additional research is needed to investigate the effects of interpersonal violence on positive sexuality and sexual assertiveness among people who are from diverse socioeconomic and cultural backgrounds. While primary prevention of sexual and physical violence victimization is essential, the prevalence rates remain relatively high. Thus, investigating approaches to mitigating the effect of an array of violence/abuse on women's current sexual health (*e.g*., positive sexuality, sexual functioning and satisfaction) proves to be an important piece of addressing health holistically.

## Supplementary Material

Supplemental data

Supplemental data

Supplemental data

## Data Sharing Statement

WHY data are available to researchers. Contact A.L.C. and K.S. for more information.

## Abbreviations Used

CA: child abuse
CSA: child sexual abuse
DF: degrees of freedom
FSSI: Female Sexual Subjectivity Inventory
HPA: hypothalamic-pituitary-adrenal
IPV: intimate partner violence
MANCOVA: multivariate analysis of covariance
NS: nonsignificant
PROMIS: Patient-Reported Outcomes Measurement Information System
PTSD: posttraumatic stress disorder
SE: standard error
SV: sexual violence
SAQ-12: Sexual Assertiveness Questionnaire
VE: variance explained
WHY: Wellness, Health and You
