# Supplementary material for: The Impacts of Lifetime Violence on Women's Current Sexual Health
Source: Womens Health Rep (New Rochelle). 2024 Feb 1;5(1):56–64. doi: 10.1089/whr.2023.0089 (PMC10890937; doi:10.1089/whr.2023.0089)
Supplement: Supplemental data [file Suppl_TableS1.docx]

**eTable1.** PROMIS Sexual Health Measures

| **Subscale** | **Questions** | **Response options** |
| --- | --- | --- |
| Sexual satisfaction (5 items, 1 factor, VE = 3.63, α = 0.903) | **How satisfied have you been with:**   - the amount of affection expressed physically in your intimate relationship? - the variety of sexual activities you engage in with your intimate partner? - your sexual relationship in general? - your overall relationship with your partner? - how satisfied do you think your partner has been with your sexual relationship in general? | not at all (=1) to very (=5) |
| Sexual interest (5 items, 1 factor VE=3.19, α=0.87) | How interested have you been in sexual activity? | not at all (=1) to very (=5) |
|  | How often have you felt like you wanted to have sex? | never (=1) to always (=5) |
|  | How would you rate your ability to have a satisfying orgasm / climax? | no sexual activity (=0) to almost always or always (=5) |
|  | When you have had sexual activity, how much have you enjoyed it? | not at all (=1) to very much (=5) |
|  | When you have had sexual activity, how satisfying has it been? | not at all (=1) to very satisfying (=5) |
| Sexual functioning (5 items, 1 factor, VE = 3.43, α = 0.78) | How often did you become lubricated ("wet") during sexual activity or intercourse? | no sexual activity (=0) to almost always or always (=5) |
|  | How difficult has it been for your vagina to get lubricated ("wet") when you wanted it to? | have not tried (=0) to not at all (=5). |
|  | How would you describe the comfort of your vagina during sexual activity?” | no sexual activity (=0) to very uncomfortable (=5) |
|  | How often have you had difficulty with sexual activity because of discomfort or pain in your vagina? | no sexual activity (=0), always (=1) to never (=5) |
|  | How often have you stopped sexual activity because of discomfort or pain in your vagina? | no sexual activity (=0), always (=1) to never (=5) |
