# Supplementary material for: The Impacts of Lifetime Violence on Women's Current Sexual Health
Source: Womens Health Rep (New Rochelle). 2024 Feb 1;5(1):56–64. doi: 10.1089/whr.2023.0089 (PMC10890937; doi:10.1089/whr.2023.0089)
Supplement: Supplemental data [file Suppl_TableS2.docx]

**eTable 2.** Female Sexual Subjectivity Inventory (FSSI)

| **Female Sexual Subjectivity Inventory (FSSI)** | | |
| --- | --- | --- |
| **Scale** | **Questions** | **Response options** |
| 1 Factor, n=1191, VE = 3.29; α = 0.928 | If a partner were to ignore my sexual needs and desires, I'd feel hurt | not sexually active (=0), strongly agree (=1) to strongly disagree (=5) |
|  | It would bother me if a sexual partner neglected my sexual needs and desires | not sexually active (=0), strongly agree (=1) to strongly disagree (=5) |
|  | I would expect a sexual partner to be responsive to my sexual needs and feelings | not sexually active (=0), strongly agree (=1) to strongly disagree (=5) |
|  | I think it is important for a sexual partner to consider my sexual pleasure | not sexually active (=0), strongly agree (=1) to strongly disagree (=5) |
