# Supplementary material for: The Impacts of Lifetime Violence on Women's Current Sexual Health
Source: Womens Health Rep (New Rochelle). 2024 Feb 1;5(1):56–64. doi: 10.1089/whr.2023.0089 (PMC10890937; doi:10.1089/whr.2023.0089)
Supplement: Supplemental data [file Suppl_TableS3.docx]

**eTable 3.** Sexual Assertiveness Questionnaire

| **Sexual Assertiveness Questionnaire** | | |
| --- | --- | --- |
| **Scale** | **Questions** | **Response options** |
| 12 items, 1 Factor, n=1191, VE = 5.58 α=0.866 | I begin sex with my partner if I want to | not sexually active (=0), strongly agree (=1) to strongly disagree (=5) |
|  | I let my partner know if I want my partner to touch my genitals | not sexually active (=0), strongly agree (=1) to strongly disagree (=5) |
|  | I wait for my partner to touch my genitals instead of letting my partner know that's what I want | not sexually active (=0), strongly agree (=1) to strongly disagree (=5) |
|  | I let my partner know if I want to have my genitals kissed | not sexually active (=0), strongly agree (=1) to strongly disagree (=5) |
|  | I give in and kiss if my partner pressures me, even if I already said no. | not sexually active (=0), strongly agree (=1) to strongly disagree (=5) |
|  | I refuse to let my partner touch my body if I don't want that, even if my partner insists | not sexually active (=0), strongly agree (=1) to strongly disagree (=5) |
|  | I have sex if my partner wants me to, even if I don't want to | not sexually active (=0), strongly agree (=1) to strongly disagree (=5) |
|  | If I said no, I won't let my partner touch my genitals even if my partner pressures me | not sexually active (=0), strongly agree (=1) to strongly disagree (=5) |
|  | I refuse to have sex if I don't want to, even if my partner insists | not sexually active (=0), strongly agree (=1) to strongly disagree (=5) |
|  | I have sex without a condom or latex barrier if my partner doesn’t like them, even if I want to use one | not sexually active (=0), strongly agree (=1) to strongly disagree (=5) |
|  | I have sex without using a condom or latex barrier if my partner insists, even if I don’t want to | not sexually active (=0), strongly agree (=1) to strongly disagree (=5) |
